# A phase III randomized clinical trial comparing laparoscopic radical hysterectomy based on open state with abdominal radical hysterectomy in patients with early-stage cervical cancer

**DOI:** 10.1186/s13063-024-08318-z

**Published:** 2024-07-11

**Authors:** Xin Zhao, Yansong Liu, Jumin Niu, Yulin Shi

**Affiliations:** Liaoning Province, Shenyang Women’s and Children’s Hospital, Shenyang City, China

**Keywords:** Cervical cancer, Laparoscopic surgery, Radical hysterectomy, Phase III clinical trial, Based on open state

## Abstract

**Background:**

Cervical cancer is the fourth most frequently diagnosed cancer and the fourth leading cause of cancer death in women, The standard treatment recommendation for women with early cervical cancer is radical hysterectomy with pelvic lymph node dissection, however, articles published in recent years have concluded that the treatment outcome of laparoscopic surgery for cervical cancer is inferior to that of open surgery. Thus, we choose a surgically new approach; the laparoscopic cervical cancer surgery in the open state is compared with the traditional open cervical cancer surgery, and we hope that patients can still have a good tumor outcome and survival outcome. This trial will investigate the effectiveness of laparoscopic cervical cancer surgery in the open-state treatment of early-stage cervical cancer.

**Method and design:**

This will be an open-label, 2-armed, randomized, phase-III single-center trial of comparing laparoscopic radical hysterectomy based on open state with abdominal radical hysterectomy in patients with early-stage cervical cancer. A total of 740 participants will be randomly assigned into 2 treatment arms in a 1:1 ratio. Clinical, laboratory, ultrasound, and radiology data will be collected at baseline, and then at the study assessments and procedures performed at baseline and 1 week, 6 weeks, and 3 months, and follow-up visits begin at 3 months following surgery and continue every 3 months thereafter for the first 2 years and every 6 months until year 4.5. The primary aim is the rate of disease-free survival at 4.5 years. The secondary aims include treatment-related morbidity, costs and cost-effectiveness, patterns of recurrence, quality of life, pelvic floor function, and overall survival.

**Conclusions:**

This prospective trial aims to show the equivalence of the laparoscopic cervical cancer surgery in the open state versus the transabdominal radical hysterectomy approach for patients with early-stage cervical cancer following a 2-phase protocol.

**Trial registration:**

ChiCTR2300075118. Registered on August 25, 2023.

**Supplementary Information:**

The online version contains supplementary material available at 10.1186/s13063-024-08318-z.

## Introduction

It is estimated that 570,000 women worldwide are diagnosed with cervical cancer every year [1]. Cervical cancer is the fourth most frequently diagnosed cancer and the fourth leading cause of cancer death in women [1]. In the past 20 years, the age-standardized incidence rate and mortality of cervical cancer in China have been rising. In 2020, 109,741 new cases and 59,060 deaths of cervical cancer, accounting for 18% and 17% of the world respectively [2]. The standard treatment recommendation for women with early cervical cancer is radical hysterectomy with pelvic lymph node dissection [3]. It is divided into laparoscopic cervical cancer surgery and traditional open cervical cancer surgery.

Previous evidence from retrospective and non-randomized studies shows that minimally invasive radical hysterectomy has better surgical effects than open radical hysterectomy, including less intraoperative bleeding, shorter hospital stay, lower postoperative incidence rate, and faster functional recovery [4–7].

However, articles published in recent years have concluded that the treatment outcome of laparoscopic surgery for cervical cancer is inferior to that of open surgery. The recently completed laparoscopic approach to cervical cancer (LACC) trial showed that in patients with early cervical cancer, the risk of death after a minimally invasive procedure is 6 times higher than that after radical hysterectomy through a traditional open incision (laparotomy). Minimally invasive procedure also has a high recurrence rate compared to open access, and the overall survival rate is lower [8]. A review of the minimally invasive approach to radical hysterectomy based on population-level data from the National Cancer Database confirmed that women with cervical cancer who undergo this surgery have worse outcomes than women with cervical cancer who have the procedure performed via laparotomy [9].

The results reported by LACC make the minimally invasive treatment of cervical cancer, a common gynecological tumor, seem to be history. Some studies showed that in the single-center study, MIS radical hysterectomy for cervical cancer does not bring a worse tumor prognosis; The 5-year DFS rate in the MIS group was 87%, while in the laparotomy group, it was 86.6% (*p* = 0.15) [10]; MIS treatment for cervical cancer can improved DFS.

Professor Chen Chunlin led and presided over the real-world study(RWS) of Big data for clinical diagnosis and treatment of cervical cancer in China [11]. Through the analysis of stage IA1(LVSI +)-IIA1 cervical cancer, they concluded that (1) in patients with stage IA (LVSI +)-IB1, when the tumor diameter is less than 2 cm, patients with any one of the high-risk factors or two or more medium risk factors requiring postoperative adjuvant treatment, the oncology outcome of laparoscopic surgery is no worse than that of open surgery. (2) Simple comparison of laparoscopic and open surgery for stage IB2 cervical cancer, laparoscopic surgery oncology outcomes are not inferior to open surgery, but must be standard treatment cases, that is, there is no preoperative adjuvant therapy, standard surgery, and postoperative adjuvant therapy. (3) The oncological outcome of laparoscopy for stage IIA1 Cervical cancer is no less than that of laparotomy. It should be said that the above results are encouraging. However, another RWS study based on the US national database was also published in NEJM during the same period as the LACC Trial study, “Survival after Minimally Invasive Radical Hysterectomy for Early-Stage Cervical Cancer” [9].

In addition, the results of the two studies are similar. Although the results are still controversial, the common results of RCT and RWS research have reached the highest level of evidence, so it is necessary to conduct extensive and in-depth research and explore why a minimally invasive procedure for cervical cancer is not like the results of laparotomy.

LACC researchers have suggested several possible reasons why surgical treatment of cervical cancer in minimally invasive surgery(MIS) is inferior to that in laparotomy: this includes the application of uterine manipulators, the establishment of pneumoperitoneum through carbon dioxide insufflation, the method of vaginal incision in vivo, and the experience of surgeons in MIS [8, 9, 12, 13]. During minimally invasive hysterectomy, uterine manipulators are often used for retraction and visualization, which may cause tumor cell proliferation and increase the tendency of tumor proliferation [8, 9]. The way of intracorporeal colpotomy in laparoscopy is considered to increase the possibility of tumor exposure to the abdominal cavity and tumor dissemination [12, 14, 15].

In solid tumor models, the current research results are uncertain whether CO_2_ pneumoperitoneum has an impact on immune suppression and tumor recurrence. In vitro studies have shown that cervical cancer cells stimulated in a CO_2_ pneumoperitoneum environment, after a short period of inhibition, have increased proliferation ability and decreased invasion, migration, and adhesion ability [16]. Clinical retrospective studies have shown that exposure to circulating CO_2_ in cervical cancer may lead to tumor spread to the abdominal cavity, with a higher recurrence rate [12, 16]. Therefore, we are not sure whether CO_2_ pneumoperitoneum is at high risk of recurrence and poor survival in patients undergoing minimally invasive radical hysterectomy, and we can take gasless pneumoperitoneum as a protective measure. Gasless laparoscopic surgery is a combination of traditional open surgery and current laparoscopic techniques, which uses physical and mechanical devices to lift the anterior abdominal wall to replace the laparoscopic surgical space built by CO_2_, with the aim of avoiding pneumoperitoneum-related complications and improving the safety of laparoscopic surgery.

Although studies have shown that minimally invasive radical hysterectomy has a worse survival rate, some surgeons may consider continuing to use this method because it may reduce the rate of surgical complications/mortality of surgery.

Therefore, based on the above research background, our surgical purpose is to reduce the possibility of tumor proliferation and tumor cell dissemination while retaining minimally invasive: (1) gasless laparoscopy surgery and abdominal wall suspension; (2) insert the port into the umbilical foramen to achieve abdominal pressure in the open state, and the smoke from the electrosurgical instrument can be smoothly discharged; (3) without the use of uterine manipulators, close the vaginal resection area before vaginal incision. The laparoscopic cervical cancer surgery in the open state is compared with the traditional open cervical cancer surgery, we hope that patients can still have a good tumor outcome and survival outcome, and a lower recurrence rate while undergoing minimally invasive procedures. Recurrence rates and characteristics, complications and incidence rate, impact on quality of life, and cost-effectiveness will also be determined.

## Patients and methods

### Design

**Fig. 1:**
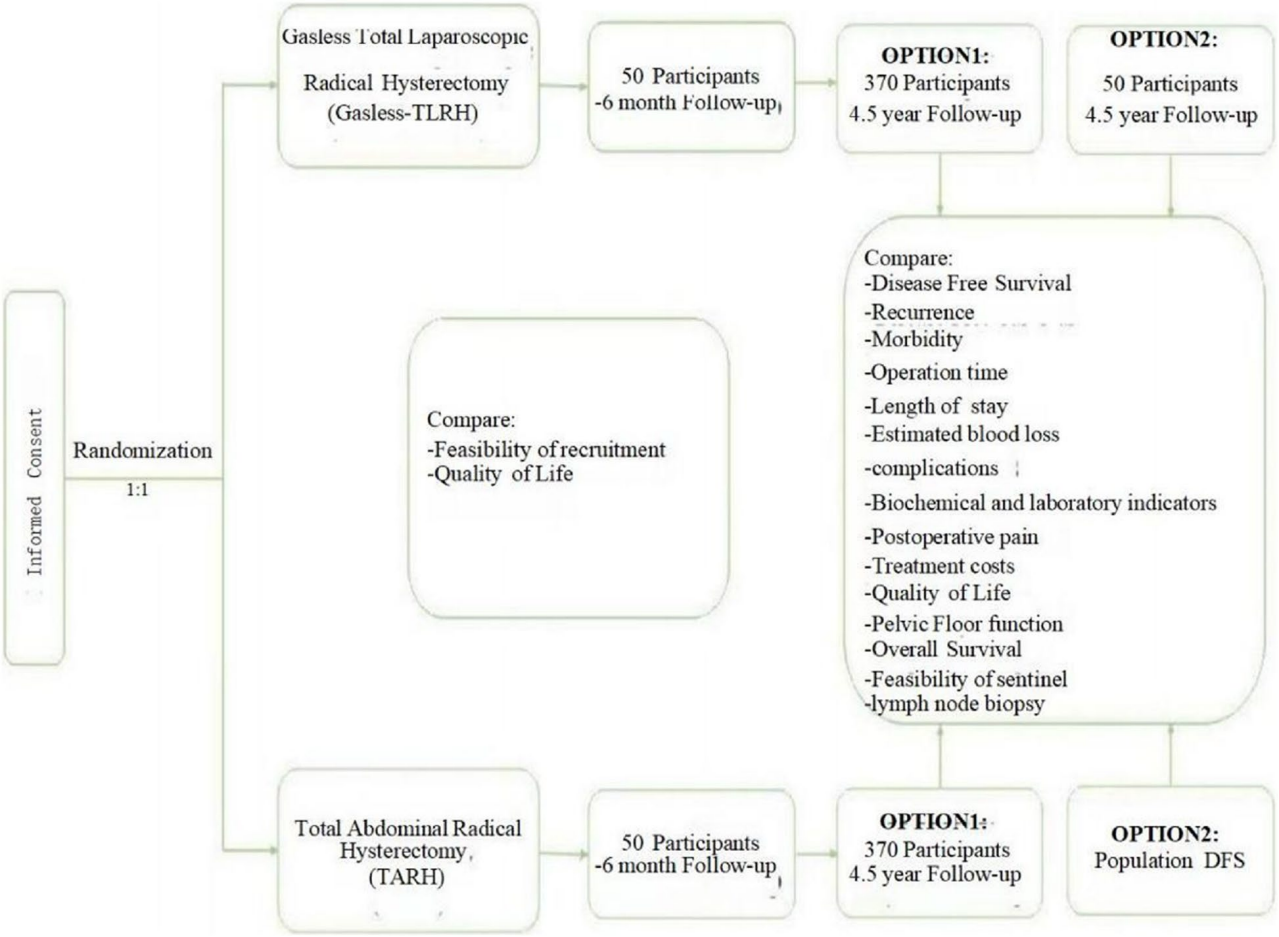
Trial design

This trial is designed as an open-label, randomized phase III trial. The study protocol was approved and registered by the Ethics Committee of Shenyang Women’s and Children’s Hospital. The purpose of randomization is to eliminate selection bias. The SPIRIT reporting guidelines were used to ensure the completion of the study protocol (Additional file 1) [17]. The study flowchart is illustrated in Fig. 1.

### Patients

Patients who meet the eligibility criteria below will be recruited from our clinic. Oral and written information about the study will be provided by the examining doctor.

### Inclusion criteria

1. Age ≥ 18 years
2. Adenocarcinoma, squamous cell carcinoma, or adenosquamous carcinoma of the cervix
3. ECOG performance status of 0–1
4. FIGO (2018) clinical stage IA1 disease with lymphovascular space invasion, IA2 disease, or IB1 disease (< 2 cm and limited to the cervix)
5. Patients could undergo either a type II or a type III radical hysterectomy (Piver classification) and pelvic lymphadenectomy
6. Postoperative adjuvant radiation therapy was recommended according to the Sedlis criteria, which are widely accepted [18]

### Exclusion criteria

1. Neuroendocrine, clear cell, or serous cell type
2. Clinically advanced disease (stages IB2–IV) with a tumor size larger than 4 cm
3. Uterine size larger than 12 cm in length
4. A history of abdominal or pelvic radiotherapy
5. Evidence of metastatic disease on positron-emission tomography–computed tomography, magnetic resonance imaging, or computed tomography
6. To be unable to undergo surgery or unable to withstand lithotomy and steep Trendelenburg position
7. Lack of compliance or living too far from a treatment center, thus not allowing adequate follow-up

### Study assessments and procedures

The study assessments and procedures were performed at baseline and 1 week, 6 weeks, 3 months, and follow-up visits begin at 3 months following surgery and continue every 3 months thereafter for the first 2 years and every 6 months until year 4.5 (Table 1) [8].

**Table 1:** Schedule of patient assessments

	Baseline (within 30 days of day 1)	Surgery	1 week	6 weeks	3 months (± 14 days)	6 months (± 14 days)	Follow-up^a^
**Assessment**							
Informed consent	√						
Medical history	√						
Physical examination	√		√	√	√	√	√
Concomitant illnesses	√		√	√	√	√	√
Concomitant medications	√		√	√	√	√	√
CT scan of pelvis/abdomen	√				√	√	√
CT scan of chest (or chest X-ray)	√				√	√	√
MRI of pelvis	√				√	√	√
Serum pregnancy test	√						
FBC	√		√	√	√	√	√
E/LFT	√		√	√	√	√	√
Hemodynamic parameters	√	√					
Blood gas analysis	√	√					
Immune factor detection;	√		√	√	√	√	
Cervical biopsy/cone biopsy	√						
Clinical staging	√						
FACT-Cx	√		√	√	√	√	√
SF-12	√		√	√	√	√	√
EQ-5D	√		√	√	√	√	√
MDASI	√		√	√	√	√	√
PFD	√					√	√
Health Services Questionnaire			√	√	√	√	√
Demographics of patients with cervical cancer	√						
Surgical treatment (gasless TLRH/TARH)		√					
Sentinel lymph node biopsy		√					
ECOG	√		√	√	√	√	√
Height	√						
Weight	√		√	√	√	√	√
BMI	√		√	√	√	√	√
Operative details		√					
Operative time, min		√					
Estimated blood loss, mL		√					
Intraoperative and postoperative complications		√					
Length of stay		√					
Pain scale (linear analog scale)			√	√	√	√	
Treatment-related morbidity			√	√	√	√	
Patient’s disease status							

E/LFT — must include creatinine, bilirubin, albumin, alkaline phosphatase (ALP), aspartate, aminotransferase (AST or SGOT), and alanine aminotransferase (ALT or SGPT)

*FBC* Full blood count, *BMI* Body mass index, *CT* Computed tomography; *ECOG* Eastern Cooperative Oncology Group, *MRI* Magnetic resonance imaging

^a^Follow-up visits begin at 9 months post-op and continue every 3 months for 2 years and every 6 months until year 4.5

### Who will take informed consent?

Researchers at participating institutions should adequately inform study participants about the trial in advance. After giving potential participants enough time to consider whether or not to participate, The investigators obtained their written informed consent.

### Additional consent provisions for the collection and use of participant data and biological specimens

We will request consent for review of participants’ medical records, and for the collection of cervical tissue to assess for cancerous cells.

## Interventions

All surgeries will be performed by the same experienced surgeon. The surgeon is a chief physician with 30 years of clinical experience, proficient in minimally invasive and open surgery techniques for early cervical cancer.

### Abdominal radical hysterectomy

Antibiotics were given 30 min before surgery; a sequential compression device (SCD) was used. Make a median incision in the abdomen, all peritoneal surfaces should be thoroughly examined, the location of any metastatic disease should be recorded in the surgical report, and a biopsy should be performed to confirm the diagnosis. The posterior pelvic peritoneum was opened, suspected enlarged lymph nodes were removed, and frozen sections were submitted for examination. If the lymph nodes are positive, the aortic lymph nodes are sampled, but radical hysterectomy will be abandoned and the patient will receive definitive chemoradiotherapy. If the pelvic lymph nodes are not suspected, radical hysterectomy (Piver type 2 or 3) and pelvic lymph node dissection are possible, and the ovaries may be removed or preserved and/or transposed. Staging of aortic lymph node dissection includes removal of lymphoid tissue to submesenteric artery tissue. The days of catheter indwelling were recorded after operation.

### The laparoscopic cervical cancer surgery in the open state

- Antibiotics were given 30 min before surgery; a sequential compression device (SCD) was used.
- To establish laparoscope operation in an open state: ① Cut a 2-cm incision in the navel and place a single port. Open the abdominal cavity, which is connected to the outside world, and the intra-abdominal pressure is consistent with the state of the open surgery. ② A 10-mm incision was made 5 cm above the umbilicus, a 10-mm trocar was punctured into the abdominal cavity, and a laparoscope was inserted. ③ A 1.0–1.2-mm steel needle was inserted subcutaneously along the white line from 4 cm above the pubic symphysis to 2 cm below the umbilicus. The suspension rod was fixed on the right side of the patient's waist, and the lifting rod crossed the white line of the abdomen. A chain with a steel needle gripper was hung on the cross bar of the suspension rod, and the suspension height of the abdominal wall was adjusted through the chain.④The auxiliary operation hole was the same as the normal laparoscopy operation hole, and the number of ports used to perform the procedure is up to the surgeon’s discretion.
- No manipulator, close the vaginectomy before incision. Specific operations are as follows:

Transvaginal sealing of cervical cancer ensures that the whole laparoscopic radical hysterectomy(LRH)operation is completed without tumor exposure. After satisfactory anesthesia, the patient took the bladder lithotomy position, performed routine disinfection, sewed the anal protective towel in the perineal water to prevent pollution, and fixed the bilateral labia minora. After vaginal disinfection again, the upper and lower vaginal hooks exposed the vagina and cancerous cervix, and the length of the vagina to be removed was determined according to the scope of lesions (usually ≥ 3 cm). Six tissue forceps were used to clamp the four walls of the vagina at the lower edge of the vaginal wall to be removed, Inject a water pad (1 mg adrenaline diluted with 500 mL normal saline) under the mucosa of the intended vaginal excision with a syringe to facilitate the separation of the vaginal wall from the bladder and rectum and reduce bleeding. Cut the vaginal wall in a circular way along the clamp of the tissue forceps with a unipolar electric knife and slightly mechanically separate it. Suture the nodules on the front and rear walls of the initially separated lower edge of the vagina to seal the cervical cancer. While pulling the suture downward, pull the hook upwards to pull the bladder and rectum, adhere to the vaginal wall along the bladder vaginal gap and rectum vaginal gap, and use sharp scissors combined with blunt fingers to further separate the vaginal wall to be removed until the bladder and rectum are folded peritoneum. At the same time, further expand the separation to both sides, reaching the knees of both ureters in the front and the bilateral uterosacral ligaments in the back. The adjacent vaginal tissues on both sides are cut off by external forceps, Suture the broken end. In order to ensure the principle of tumor free to the greatest extent, rinse the vagina with saline again and collect the washing solution for tumor cytology. After the second iodophor disinfection, the front and back of the vagina are filled with gauze to reach the front and back of the retroflex peritoneum, and then start the laparoscopy. The fundus sutures were pulled instead of lifting the uterus. The absorbable line No. 1 was used to perform Fig. 8 sutures on the fundus. The intraoperative assistant clamps the suture line to swing the uterus to avoid the use of lifting the uterus.

- All peritoneal surfaces should be thoroughly examined, the location of any metastatic disease should be recorded in the surgical report, and a biopsy should be performed to confirm the diagnosis. The posterior pelvic peritoneum was opened, suspected enlarged lymph nodes were removed, and frozen sections were submitted for examination. If the lymph nodes are positive, the aortic lymph nodes are sampled, but radical hysterectomy will be abandoned and the patient will receive definitive chemoradiotherapy. If the pelvic lymph nodes are not suspected, radical hysterectomy (Piver type 2 or 3) and pelvic lymph node dissection are possible, and the ovaries may be removed or preserved and/or transposed. Staging of aortic lymph node dissection includes removal of lymphoid tissue to submesenteric artery tissue. Closure technique for the vaginal vault is left to the discretion of the surgeon; Alternatively, the laparoscopic procedure may be completed vaginally.

### Adjuvant radiotherapy

Findings at surgery are used to determine the need for adjuvant postoperative treatment. For this study, criteria [13] (Table 2) will be reviewed in making recommendations for adjuvant radiotherapy. The delivery and management of radiation therapy will be based on local institutional clinical practice guidelines. Preliminary and final dosimetry information and concurrent administration of chemotherapy will be recorded [8].

**Table 2:** Criteria for postoperative radiotherapy

CLS	Stromal invasion	Tumor size
Positive	Deep 1/3	Any
Positive	Middle 1/3	≥ 2cm
Positive	Superficial 1/3	≥ 5cm
Negative	Deep or middle 1/3	≥ 4cm

*CLS* Capillary lymphatic space tumor involvement

### Randomization

Patients will be randomly assigned to arm A (gasless TLRH) or arm B(TARH) in a 1:1 ratio by an internet-based distant third-party statistician blinded to the study and participant details. The doctor involved in recruitment will receive training and instructions on the recruitment procedure [18]. Only in case of a serious adverse event unblinding is permissible.

### Treatment of adverse events

Any adverse events will be managed by local investigators according to current good clinical practice guidelines. When the investigator realizes that a serious illness or other adverse events are found in the course of the clinical study, the investigator should report it to the DMC and relevant regulatory bodies as required indicating expectedness, seriousness, severity, and causality. The details of each adverse event will be described in a case report form, including the nature of the adverse event, time of onset and time of resolution, severity, treatment, and outcome. If necessary, a follow-up examination will be performed to ensure patient safety. If the physician monitoring the trial finds evidence of harm to a participant or signs of ineffectiveness, the participant will be withdrawn from the study. The results of these participants will be analyzed as a non-pCR group [18].

### Criteria for discontinuation of trial treatment

The criteria for the discontinuation of a trial medication are as follows:

1. A participant declines further participation or withdraws their consent.
2. Cancelation of the entire study.
3. Death.
4. The protocol treatment will be stopped if the annual patient accrual is less than 30, less than 75% of patients are available for follow-up, or an unacceptable rate of the incidence of complications (8%) occurs in the gasless TLRH group.
5. Severe adverse events (progressive or persistent) which may be related to the surgery (such as severe surgical complications, fistula, for example, occur more than 3%) and newly diagnosed other malignancies (e.g., breast cancer, excluded transfer) will be evaluated by 2 chief physicians before the trial is stopped.
6. Any situation in which the treatment cannot be continued according to the judgment of the physician [19].

### Discontinuation of the study

The study will be terminated early if the IRB determines the occurrence of any of the following: serious surgical complications (fistula); the participants with unexpected, significant, or unacceptable risks (such as death); the trial treatment is determined to be ineffective [19].

### Baseline assessments

Baseline assessments will be performed according to the trial standard operating procedure (SOP), including medical history, determination of BMI, CT and MRI imaging, blood tests, and cervical tissue specimens will be obtained by treating gynecologic oncologists. Histopathological examination of tissue specimens will confirm the cell type and grade of differentiation [19].

### Efficacy assessments

#### The primary outcome is the rate of disease-free survival at 4.5 years

To compare disease-free survival amongst patients who undergo laparoscopic cervical cancer surgery based on open surgery versus those who undergo a total abdominal radical hysterectomy (TARH) for early-stage cervical cancer [8].

#### Secondary outcome measures (compare between arms)

Operation time: The time from the start of the operation to the end of the operation.

Length of stay: The time from admission to discharge.

Estimated blood loss. Blood loss will be defined as the total volume of suctioned fluids minus the volumes of irrigation fluids used at the completion of surgery.

The levels of CD4^+^ T lymphocytes, NK cells, and CD4^+^ CD25^high^ CD127^low^ Treg in peripheral blood before and after operation; postoperative peritoneal immune factors IL-6, TNF-β, and TNF-α levels.

Hemodynamic parameters (cardiac output before and at the end of surgery, CO), mean arterial pressure (MAP), and stroke output (stroke volume (SV)). Pco2, blood gas analysis, ion profile.

Intraoperative complications (injury to bladder, ureters, bowel, blood vessels and bleeding, and nerves).

Perioperative complications: urinary tract infection, urinary retention, ileus, myocardial infarction, atrial fibrillation, pulmonary edema, atelectasis, pneumonia, renal and cerebrovascular morbidity, and thromboembolic complications (deep vein thrombosis, pulmonary embolism).

Early (4 weeks) postoperative complications: pulmonary, renal, and cerebrovascular morbidity; wound and vault complications (infection, breakdown, and dehiscence); septicemia and thromboembolic complications (deep vein thrombosis, pulmonary embolism); and lymphocyst or abscess formation.

Delayed postoperative complications (4 weeks–6 months after surgery): lymphedema, incisional hernia formation, vaginal evisceration.

Postoperative pain and analgesic consumption using pain scores and analgesic consumption measurements.

Costs: measured as the incremental cost/unit of improvement in functional outcome, measured in terms of the primary outcome plus using quality-adjusted life years to undertake a cost-use analysis.

The quality of life instruments(QOL) (FACT-Cx, SF12, EQ-5D, MDASI): change in QOL using functional assessment of cancer therapy-cervical between baseline (presurgery) and 6 months after surgery.

Pelvic floor distress inventory: measures symptom severity and QOL changes in women with pelvic floor disorders. The pelvic floor distress inventory provides a standardized, reproducible assessment of the patient’s symptoms and their effect on daily life.

The OS at 4.5 years follow-up [8].

### Oversight and monitoring

#### Composition of the coordinating center and trial steering committee

Prior to the start of the study, the gynecologic medical team will receive formal training about the study protocol and data collection procedures. All necessary research documents will be made available to each researcher at any time. Medical researchers will supervise the screening and inclusion of patients.

The trial’s day-to-day coordination will be managed by two gynecologists and one pathologist, along with support from an epidemiologist, Trial Steering Committee overall management, monitoring, auditing, data management, statistical analysis, and writing, regularly submit progress report results twice a year.

#### Data monitoring committee

The data monitoring committee (DMC) will be composed of clinical trial specialists, including a biostatistician, who are not associated with the study. The committee will meet at least twice a year, and all data obtained from the trial will be evaluated by the committee. The DMC will review unblinded outcome data for safety and efficacy, and judge if there is evidence that either treatment is unsafe and the trial should be discontinued. The DMC will also advise the Trial Steering Committee of any evidence of unethical treatment or unacceptably serious adverse events. The IBM Clinical Development Management System (IBM Corporation, Somers, New York), which is based on an EDC (Electronic Data Capture System), will be used to manage the data in this study [19].

### Sample size

The main purpose of this study is to determine whether gasless laparoscopic radical hysterectomy (gasless TLRH) or equivalent to total abdominal hysterectomy (TARH) is effective in terms of disease-free survival at 4.5 years. If the difference in disease-free survival rate does not exceed 7%, then these two surgeries are considered equivalent.

The estimated 4.5-year disease-free survival rate of patients receiving TARH is 90%. The sample size was based on an expected disease-free survival rate of 90% in the open-surgery group at 4.5 years and a noninferiority margin of 7.2 percentage points for minimally invasive surgery, which reflected an acceptable difference in the expected survival rate of at most 8 percentage points. In previous studies involving patients with other types of cancer, a noninferiority margin of 6 to 8 percentage points has been considered to be clinically acceptable.

Based on these data, a total of 740 patients (370 in each group) will be sufficient to declare equivalence in 4.5 years of accrual and 4.5 years of follow-up, with an equivalence margin of 6% or less at 4.5 years [8].

### Recruitment

Eligible patients will be continuously recruited until the target sample size is reached. Investigators of participating hospitals identified potential trial participants from routine medical practice. All outpatients were asked if they wanted to participate when they were told that they were getting an operation. We will recruit 740 participants over 5 years.

### Statistical analysis

The main endpoint will be analyzed based on the intention-to-treat principle.

#### Phase 1 analysis (feasibility pilot)

After accumulating 100 patients, the data will be analyzed to determine several key points and analysis of surgical safety.

A research component that is not the primary endpoint specified in the protocol. These will include:

- Accrual rate
- Compliant with randomized treatment allocation

If these components are satisfactory, the Test Management Committee will make a decision to proceed.

In the second stage, 740 patients will receive complete therapy.

#### Phase II analysis (phase III study)

The disease-free survival and overall survival curves will be estimated using the Kaplan–Meier method. The main comparison of survival distribution will be compared with the logrank test. The secondary analysis will use appropriate regression models (such as Cox proportional risk models) to adjust for prognostic factors. All efficacy indicators will be compared through intention-to-treat analysis, including all randomized patients. The toxicity will be analyzed based on the treatment received. The difference between treatment groups will be reported with a 95% confidence interval.

Descriptive statistics on treatment-related adverse events and quality of life (FACT-Cx, SF-12). At each evaluation, subscales (Physical Health, Social Health, Emotional Health, Functional Health, Cervical Cancer Specific Health, and Body Image Scale) will be calculated for each random group.

Similarly, descriptive statistics will be calculated for other results, such as pain score, anxiety and depression score, Analgesic consumption, etc.

The normality of continuous variables and the equality of inter-group variance will be evaluated.

Discrete variables (such as the presence/absence of postoperative infection) will be summarized as follows frequency/proportion.

For continuous variables, analysis of variance and/or regression analysis will be used where appropriate. If the assumptions of these tests are violated, alternative non-parametric tests will be used. Differences between groups with respect to discrete variables will be assessed using the chi-squared test.

Exploratory analysis adjusts prognostic factors including age, tumor size, staging, and tumor grading.

Differentiation, depth of muscle invasion, lymph node involvement, treatment type, and ECOG status will be evaluated using proportional risk regression methods. The impact of baseline quality of life on survival will also be studied.

We will analyze the results related to treatment costs based on different surgical methods [8].

### Methods for additional analyses (e.g., subgroup analyses)

Subgroup analyses will not be conducted. However, we will apply sensitivity analysis of per-protocol analysis to assess potential biases.

## Discussion

This trial is expected to provide high-level evidence that laparoscopic radical hysterectomy with a closed cervical cancer body, no lifting of the uterus, and no pneumoperitoneum may be a valid alternative to abdominal surgery with equal DFS and OS and superior short-term well-being for women diagnosed with early-stage cervical cancer.

It is reported that the continuously perfusing and flowing carbon dioxide in the abdominopelvic cavity could lead to the spread of the detached tumor cells [12, 13, 16]. Gasless laparoscopic surgery (GLS) is an effective operation in the treatment of gynecological diseases with few complications [20].

Investigated the usefulness of GLS using a subcutaneous abdominal wall lifting method for endometrial cancer.GLS for endometrial cancer results in less bleeding, shorter hospital stay, and fewer complications than open surgery. Recurrence and survival rates were not significantly different from those of open surgery [21]. Regardless of whether the CO_2_ pneumoperitoneum environment increased tumor cell growth or spread, our gasless laparoscopy was similar to laparotomy, which may be a protective measure. In the space formed by the suspension of the abdominal wall, the abdominal pressure is consistent with atmospheric pressure, which has little influence on the movement of the diaphragm, reduces the inflow of CO_2_ into the blood, and affects the blood gas. Eliminating the effects and risks related to CO_2_ has higher safety and is less likely to cause significant reactions to the respiratory and circulatory systems.

Andreas Kavallaris et al. retrospective 32 patients with stage FIGO (2009) IB cervical cancer underwent laparoscopic radical hysterectomy without the use of any uterine manipulator. This study showed a better tumor prognosis compared to studies using a minimally invasive approach with routine use of uterine manipulators and no vaginal closure of tumors [22]. In 2020, a large-scale cohort observation study SUCCOR showed that MIRH using different techniques (including avoiding uterine manipulators) yielded comparable results to existing techniques [23]. Therefore, compared to open surgery, cancer cell leakage may lead to poorer tumor prognosis.

All reported studies are retrospective designs, and most have not adjusted for confounding factors. In the future, high-quality prospective studies must be considered to validate the skills of surgeons. There are currently two ongoing clinical trials, including JGOG1087, a non-randomized controlled LRH trial in Japan aimed at preventing cancer cell overflow, and SOLUTION, a phase 2 non-inferiority trial evaluating the oncological outcomes of MIS using endoscopic sutures as a tool for preventing cancer cell overflow [24].

Retrospective case series on the laparoscopic treatment of early cervical cancer without pneumoperitoneum and uterine manipulator have been published. In addition, a number of prospective, randomized, and non-randomized clinical trials of the treatment of cervical cancer with different surgical pathways are ongoing. Trial NCT04999696 (ClinicalTrial.gov) is Minimally Invasive Therapy Versus Open Radical Hysterectomy for Management of Early Stage Cervical Cancer. This study aims to compare the differences in disease-free survival and overall survival between laparoscopic total hysterectomy and open total hysterectomy. Recurrence rates and characteristics, complications and incidence rate, impact on quality of life and cost-effectiveness will also be determined.820 people were included, covering the period from 2023 to 2033. Trial NCT03955185 is Clinical Trial of Minimally Invasive Surgery Versus Abdominal Surgery in Patients With Early Stage Cervical Cancer (RWS-01). They plan to recruit 2000 early cervical cancer patients from 20 to 30 selected surgical hospitals nationwide and have qualified and experienced doctors perform surgeries on the patients. They will provide detailed information on the patient's current research status and divide them into different observation groups based on their choice of surgical method. Close follow-up will be conducted on patients after surgery. They will compare the clinical outcomes of the two surgical methods and conduct subgroup and stratified analyses. We hope that this study can truly reflect the actual situation and clinical level of early cervical cancer treatment in China, and provide high-level clinical evidence for the treatment of cervical cancer in China.NCT04939831、NCT04929769、NCT04934982 through a multicenter stratified randomized controlled study, comparing different stages of cervical cancer, laparoscopic or abdominal radical hysterectomy for PFS and OS of patients with early cervical. Explore whether stricter surgical detail specifications (including tumor-free principles and standard surgical scope) can improve the PFS and OS of LRH, evaluate postoperative complications, and quality of life.

This proposed trial has strengths and weaknesses. A marked strength is that no high-quality studies are comparing the efficacy of gasless laparoscopic or abdominal surgery in the treatment of early cervical cancer. The current research experiments include ChiCTR2200064194, vaginal assisted single port laparoscopic radical surgery for cervical cancer using pneumoperitoneum and non-pneumoperitoneum: a prospective randomized controlled study. ChiCTR20000376, a randomized controlled study on the efficacy and safety of transumbilical single-hole laparoscopic surgery and traditional multi-hole laparoscopic surgery for cervical cancer. ChiCTR2100045060, a prospective randomized controlled study of robot and laparoscopic radical cervical cancer surgery. At present, there is no comparison of the clinical efficacy, safety, and clinical value between gasless laparoscopy and open surgery. If the outcome of gasless laparoscopy is the same as or better than open surgery, so this procedure had the advantages of all minimally invasive approaches, such as fast recovery and esthetic advantages. Strengths of our work also include its prospective design, standardized treatment protocol, and long duration of follow-up.

A limitation of this trial is that the design is not that of a confirmatory trial. Evaluation of the overall survival rate and recurrence rates, which are associated with the primary endpoint, will be performed by a clinical review board. The evaluation will be conducted under blind and independent conditions. And we will apply sensitivity analysis of per-protocol analysis to assess potential biases. Therefore, we believe that it will be possible to maintain objectivity and reduce potential bias. In addition, this is a single-center, randomized controlled trial design, although the operation is easy to operate and can be widely applied for gynecologists who have the foundation of traditional open cervical cancer surgery and laparoscopic surgery, in the future, we need multicenter, prospective studies to draw further strong conclusions.

We hope that the current study will increase the level of evidence on the comparison of tumor outcomes between gasless laparoscopy and open cervical surgery. We hope that “Laparoscopic radical hysterectomy with closed cervical cancer body and no lifting of uterus and no pneumoperitoneum,” that is, laparoscopy based on the open status has an encouraging survival outcome in the treatment of early cervical cancer, while improving the quality of life of patients. It is necessary to scientifically prove the efficacy of these surgical procedures in high-quality studies. We believe that the results of these studies will be important for the future direction of minimally invasive surgery.

### Trial status

This study protocol was approved on May 2023. The current protocol is version 1. The first patient is planned to be randomized on September 2023 and the expected end of recruitment is October 2028.

### Supplementary Information

Additional file 1. SPIRIT checklist. 

## Data Availability

Requests for data generated during this study will be considered by DMC. Data will typically be available within 6 months after the primary publication. Only scientifically sound proposals from appropriately qualified Research Groups will be considered for data sharing. The request will be reviewed by the Data Sharing Committee in discussion with the Chief Investigator and, where appropriate (or in the absence of the Chief Investigator) any of the following: the Trial Sponsor, the relevant Trial Management Group (TMG), and independent Trial Steering Committee (TSC). A formal Data Sharing Agreement (DSA) may be required between respective organizations once the release of the data is approved and before data can be released. Data will be fully deidentified (anonymized) unless the DSA covers the transfer of patient identifiable information. Any data transfer will use a secure and encrypted method. The authors promise to provide the original data supporting this study without reservation (provided by Yulin Shi, email: 15,040,291,279@163.com).

## Abbreviations

DFS: Disease-free survival
OS: Overall survival
MIS: Minimally invasive surgery
LACC: Laparoscopic Approach to Cervical Cancer
SCD: Sequential compression device
LRH: Laparoscopic radical hysterectomy
SOP: Standard operating procedure
QOL: Quality of life instruments
DMC: Data monitoring committee
EDC: Electronic Data Capture System
TARH: Total abdominal hysterectomy
RWS: Real-world study
GLS: Gasless laparoscopic surgery
